# Surveillance of Omadacycline Activity Tested against Clinical Isolates from the United States and Europe as Part of the 2016 SENTRY Antimicrobial Surveillance Program

**DOI:** 10.1128/AAC.02327-17

**Published:** 2018-03-27

**Authors:** Michael A. Pfaller, Michael D. Huband, Dee Shortridge, Robert K. Flamm

**Affiliations:** a JMI Laboratories, North Liberty, Iowa, USA; b University of Iowa, Iowa City, Iowa, USA

**Keywords:** omadacycline

## Abstract

Omadacycline was tested against 21,000 bacterial isolates collected prospectively from medical centers in Europe and the United States during 2016. Omadacycline was active against Staphylococcus aureus (MIC_50_/MIC_90_, 0.12/0.25 mg/liter), including methicillin-resistant S. aureus (MRSA); streptococci (MIC_50_/MIC_90_, 0.06/0.12 mg/liter), including Streptococcus pneumoniae, viridans group streptococci, and beta-hemolytic streptococci; Enterobacteriaceae, including Escherichia coli (MIC_50_/MIC_90_, 0.5/2 mg/liter); Haemophilus influenzae (MIC_50_/MIC_90_, 1/1 mg/liter); and Moraxella catarrhalis (MIC_50_/MIC_90_, 0.25/0.25 mg/liter). Omadacycline merits further study in serious infections where resistant pathogens may be encountered.

## TEXT

Tetracyclines are broad-spectrum antibacterial agents that also possess activity against intracellular pathogens, protozoans, and helminthic parasites (1). Tetracycline use has resulted in the emergence of strains that are resistant (R) to tetracycline, which limits the use of the older members of this class (1, 2). Omadacycline is a novel aminomethylcycline (3–5) that binds to the 30S ribosomal subunit of target bacteria, resulting in protein synthesis inhibition (1, 3, 4). Omadacycline retains activity against tetracycline-resistant bacterial strains expressing both ribosomal protection and efflux resistance genes (2, 4, 5). Omadacycline maintains activity against difficult-to-treat pathogens, including methicillin-resistant Staphylococcus aureus (MRSA), vancomycin-resistant enterococci (VRE), Enterobacteriaceae that produce extended-spectrum β-lactamases (ESBLs) and carbapenemases, and multidrug-resistant (resistant to ≥3 classes of agents) strains of Acinetobacter spp. and Stenotrophomonas maltophilia (2). Omadacycline has shown noninferiority to linezolid in both an intravenous-to-oral switch, phase 3 acute bacterial skin and skin structure infection (ABSSSI) study and an oral-only ABSSSI study, as well as noninferiority to moxifloxacin in an intravenous-to-oral switch, community-acquired bacterial pneumonia (CABP) study (6–8).

Bacterial isolates (*n* = 21,000) were collected prospectively from hospitalized patients in 68 medical centers in the United States and Europe for the 2016 SENTRY Antimicrobial Surveillance Program. Isolate identifications were established by the participating medical centers and confirmed at JMI Laboratories (North Liberty, IA), when necessary. Omadacycline MIC values were determined using the reference Clinical and Laboratory Standards Institute (CLSI) broth microdilution method (9). Quality control (QC) and interpretation of results were performed in accordance with CLSI document M100-S27 and European Committee on Antimicrobial Susceptibility Testing (EUCAST) 2017 guidelines (10, 11). Enterobacteriaceae isolates were classified as susceptible (S) to ceftazidime (MIC, ≤4 mg/liter), nonsusceptible (NS) to ceftazidime (MIC, ≥8 mg/liter), R to imipenem (MIC, ≥4 mg/liter), R to tetracycline (MIC, ≥16 mg/liter), and NS to tigecycline (MIC, >2 mg/liter). Other resistant phenotypes included MRSA (oxacillin MIC ≥ 4 mg/liter or cefoxitin MIC > 8 mg/liter), vancomycin-NS enterococci (MIC ≥ 8 mg/liter), tetracycline-R A. baumannii, staphylococci, enterococci (all MIC ≥ 16 mg/liter), and Streptococcus pneumoniae (MIC ≥ 4 mg/liter), macrolide-R S. pneumoniae (erythromycin MIC ≥ 1 mg/liter and azithromycin MIC ≥ 2 mg/liter), and macrolide-R β-hemolytic streptococci (BHS) (erythromycin MIC ≥ 1 mg/liter). Haemophilus influenzae isolates were divided into β-lactamase-positive and -negative groups. QC strains were tested concurrently and included Escherichia coli ATCC 25922 and ATCC 35218, S. aureus ATCC 29213, Pseudomonas aeruginosa ATCC 27853, Enterococcus faecalis ATCC 29212, and S. pneumoniae ATCC 49619. All QC results, including all omadacycline MIC values, were within published ranges.

Resistance phenotype frequencies were as follows: 34.1% of S. aureus isolates were methicillin R, 63.6% of coagulase-negative staphylococcus (CoNS) isolates were methicillin R, 40.0% of Enterococcus faecium isolates were vancomycin NS, 11.6% of S. pneumoniae isolates were penicillin R, 31.4% of S. pneumoniae isolates were macrolide R, 14.4% of E. coli isolates were ceftazidime NS, 28.6% of Klebsiella pneumoniae isolates were ceftazidime NS, 27.9% of Enterobacter cloacae species complex (SC) isolates were ceftazidime NS, and 8.1% of K. pneumoniae isolates were imipenem R (Tables 1 and 2). A total of 4,890 isolates were tetracycline R, including 5.2% of S. aureus, 12.7% of CoNS, 69.4% of E. faecalis and E. faecium, 20.0% of S. pneumoniae, 34.3% of viridans group streptococcus (VGS), 43.6% of BHS, 32.8% of Enterobacteriaceae, 66.4% of Acinetobacter baumannii, 0.2% (data not shown) of H. influenzae, and 11.1% (data not shown) of Haemophilus parainfluenzae (Tables 1 and 2) isolates.

**TABLE 1:**
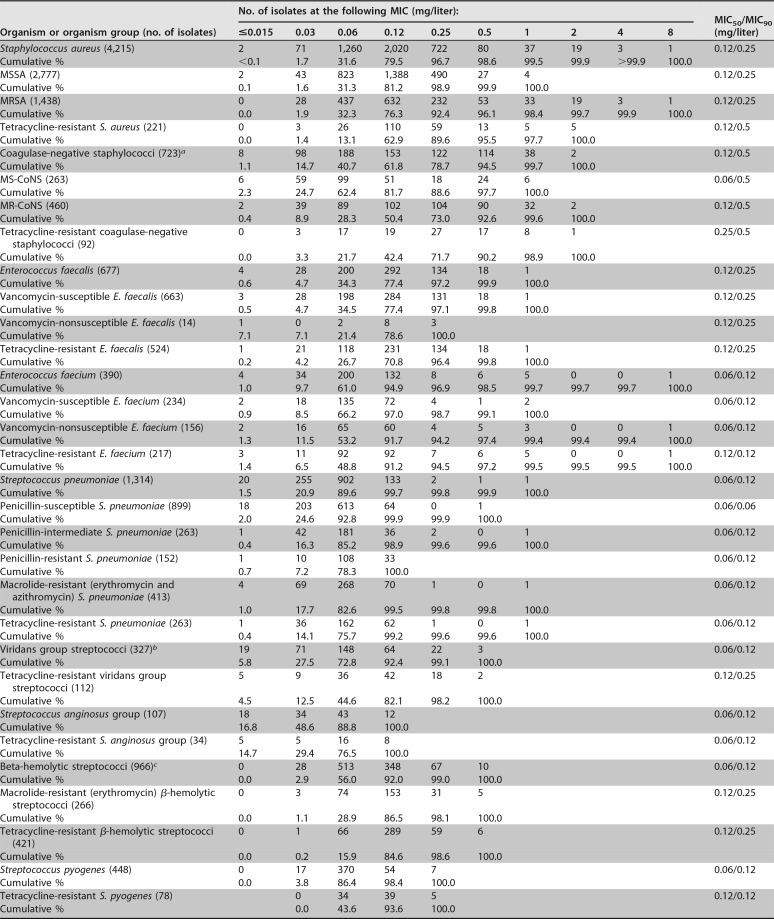

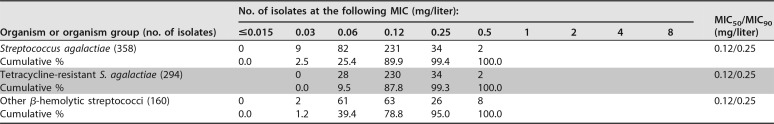
Antimicrobial activities of omadacycline againstthe main organisms and organism groups of Gram-positive cocci

a Organisms include the following Staphylococcus species: S. capitis (*n* = 45), S. caprae (*n* = 8), S. cohnii (*n* = 6), S. epidermidis (*n* = 400), S. haemolyticus (*n* = 75), S. hominis (*n* = 71), S. lugdunensis (*n* = 64), S. pettenkoferi (*n* = 9), S. saprophyticus (*n* = 18), S schleiferi (*n* = 5), S. simulans (*n* = 8), S. warneri (*n* = 13), and a coagulase-negative staphylococcus not identified to the species level (*n* = 1).

b Organisms include the following Streptococcus species or group: S. anginosus (*n* = 67), S. anginosus group (*n* = 20), S. australis (*n* = 3), S. bovis group (*n* = 2), S. constellatus (*n* = 14), S. cristatus (*n* = 3), S. equinus (*n* = 1), S. gallolyticus (*n* = 24), S. gordonii (*n* = 7), S. infantis (*n* = 1), S. intermedius (*n* = 6), S. lutetiensis (*n* = 4), S. massiliensis (*n* = 1), S. mitis group (*n* = 112), *S. mitis/S. oralis* (*n* = 2), S. mutans (*n* = 2), S. parasanguinis (*n* = 23), S. salivarius (*n* = 5), S. salivarius group (*n* = 4), *S. salivarius/S. vestibularis* (*n* = 9), S. sanguinis (*n* = 11), and S. vestibularis (*n* = 6).

c Organisms include Streptococcus agalactiae (*n* = 358), Streptococcus canis (*n* = 4), Streptococcus dysgalactiae (*n* = 156), and Streptococcus pyogenes (*n* = 448).

**TABLE 2:**
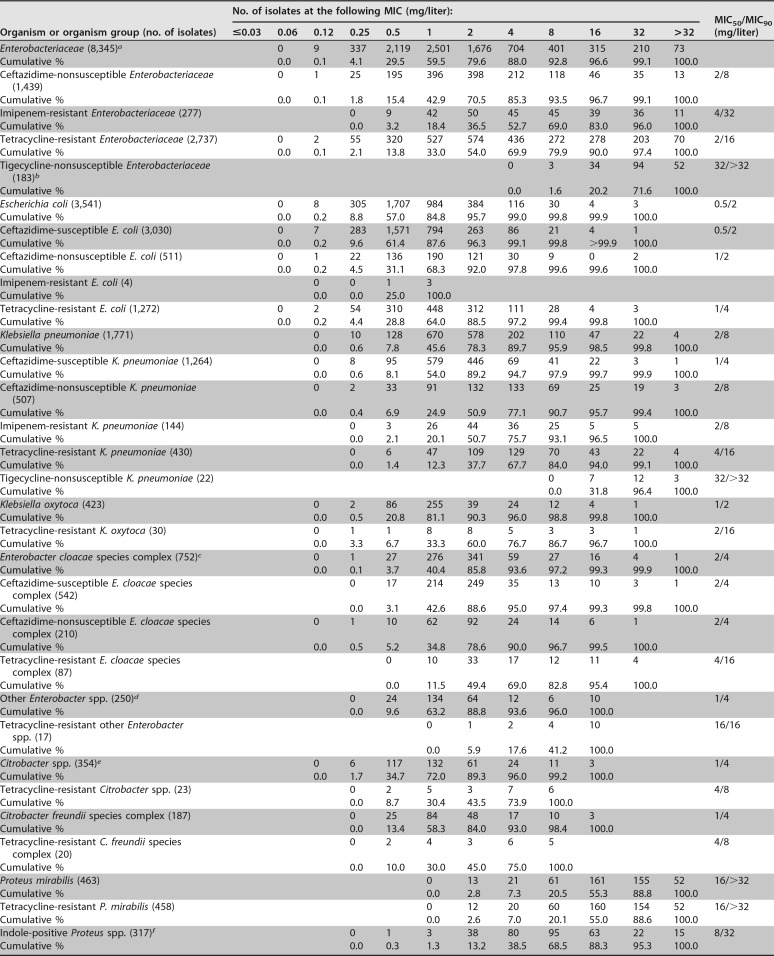

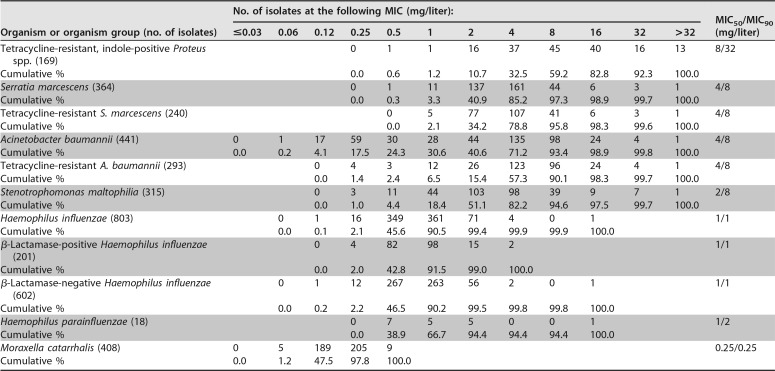
Antimicrobial activity of omadacycline tested against the main organisms and organism groups of Gram-negative bacilli

a Organisms include the following: Citrobacter amalonaticus (*n* = 5), *Citrobacter amalonaticus/Citrobacter farmeri* (*n* = 3), Citrobacter braakii (*n* = 5), Citrobacter farmeri (*n* = 3), Citrobacter freundii (*n* = 86), Citrobacter freundii species complex (*n* = 92), Citrobacter koseri (*n* = 156), Citrobacter sedlakii (*n* = 2), Citrobacter youngae (*n* = 2), Enterobacter aerogenes (*n* = 248), Enterobacter amnigenus (*n* = 2), Enterobacter asburiae (*n* = 9), Enterobacter cloacae (*n* = 368), Enterobacter cloacae species complex (*n* = 373), Enterobacter kobei (*n* = 2), Escherichia coli (*n* = 3,541), Gram-negative rods in the family Enterobacteriaceae (*n* = 2), Hafnia alvei (*n* = 24), Klebsiella oxytoca (*n* = 423), Klebsiella pneumoniae (*n* = 1,771), Kosakonia cowanii (*n* = 1), Leclercia adecarboxylata (*n* = 5), Morganella morganii (*n* = 175), Pantoea agglomerans (*n* = 9), Pantoea calida (*n* = 2), Pluralibacter gergoviae (*n* = 4), Proteus mirabilis (*n* = 463), Proteus penneri (*n* = 5), Proteus vulgaris (*n* = 2), Proteus vulgaris group (*n* = 58), Providencia alcalifaciens (*n* = 1), Providencia rettgeri (*n* = 38), Providencia stuartii (*n* = 41), Rahnella aquatilis (*n* = 1), Raoultella ornithinolytica (*n* = 17), Raoultella planticola (*n* = 4), Serratia liquefaciens (*n* = 15), Serratia marcescens (*n* = 364), Serratia odorifera (*n* = 1), Serratia rubidaea (*n* = 1), Cedecea organism not identified to the species level (*n* = 1), Pantoea organisms not identified to the species level (*n* = 7), Providencia organisms not identified to the species level (*n* = 2), Raoultella organisms not identified to the species level (*n* = 8), Serratia organisms not identified to the species level (*n* = 2), and Yersinia enterocolitica (*n* = 1).

b Organisms include the following: Enterobacter aerogenes (*n* = 1), Enterobacter cloacae species complex (*n* = 6), Escherichia coli (*n* = 2), Klebsiella pneumoniae (*n* = 22), Morganella morganii (*n* = 2), Proteus mirabilis (*n* = 140), Providencia rettgeri (*n* = 1), Providencia stuartii (*n* = 6), and Serratia marcescens (*n* = 3).

c Organisms include the following: Enterobacter asburiae (*n* = 9), Enterobacter cloacae (*n* = 368), Enterobacter cloacae species complex (*n* = 373), and Enterobacter kobei (*n* = 2).

d Organisms include the following: Enterobacter aerogenes (*n* = 248) and Enterobacter amnigenus (*n* = 2).

e Organisms include the following Citrobacter species: C. amalonaticus (*n* = 5), *C. amalonaticus/C. farmeri* (*n* = 3), C. braakii (*n* = 5), C. farmeri (*n* = 3), C. freundii (*n* = 86), C. freundii species complex (*n* = 92), C. koseri (*n* = 156), C. sedlakii (*n* = 2), and C. youngae (*n* = 2).

f Organisms include the following: Morganella morganii (*n* = 175), Proteus vulgaris (*n* = 2), Proteus vulgaris group (*n* = 58), Providencia alcalifaciens (*n* = 1), Providencia rettgeri (*n* = 38), Providencia stuartii (*n* = 41), and Providencia organisms not identified to the species level (*n* = 2).

Omadacycline MIC distributions are shown in Tables 1 and 2. Against S. aureus (*n* = 4,215) (MIC_50_/MIC_90_, 0.12/0.25 mg/liter), omadacycline inhibited 99.9% of isolates at ≤2 mg/liter, including 100.0% of methicillin-S S. aureus (MSSA) and 99.7% of MRSA isolates (Table 1). All CoNS isolates were susceptible to omadacycline at ≤2 mg/liter (MIC_50_/MIC_90_, 0.12/0.5 mg/liter). Tetracycline resistance had little effect on omadacycline MIC values against S. aureus (MIC_50_/MIC_90_, 0.12/0.5 mg/liter) or CoNS (MIC_50_/MIC_90_, 0.25/0.5 mg/liter) isolates (Table 1 and see Table S1 in the supplemental material). Omadacycline was slightly more active against E. faecium (MIC_50_/MIC_90_, 0.06/0.12 mg/liter) than against E. faecalis (MIC_50_/MIC_90_, 0.12/0.25 mg/liter) and was not adversely affected by vancomycin or tetracycline resistance (Tables 1 and S1). Omadacycline potencies were comparable for S. pneumoniae (MIC_50_/MIC_90_, 0.06/0.12 mg/liter), VGS (MIC_50_/MIC_90_, 0.06/0.12 mg/liter), and BHS (MIC_50_/MIC_90_, 0.06/0.12 mg/liter) isolates, regardless of species and susceptibility to penicillin, macrolides, or tetracycline (Tables 1 and S1). Omadacycline showed useful activity against most Enterobacteriaceae isolates (MIC_50_/MIC_90_, 1/8 mg/liter; 88.0% were inhibited at ≤4 mg/liter) (Table 2) except Proteus mirabilis (MIC_50_/MIC_90_, 16/>32 mg/liter), indole-positive Proteus spp. (MIC_50_/MIC_90_, 8/32 mg/liter), and tigecycline-NS K. pneumoniae (MIC_50_/MIC_90_, 32/>32 mg/liter) (Tables 2 and S2). Omadacycline was most active against E. coli (MIC_50_/MIC_90_, 0.5/2 mg/liter) (Tables 2 and S2). Omadacycline activity was slightly greater against ceftazidime-S than against ceftazidime-NS strains of E. coli (MIC_50_s/MIC_90_s, 0.5/2 mg/liter and 1/2 mg/liter, respectively) and K. pneumoniae (MIC_50_s/MIC_90_s, 1/4 mg/liter and 2/8 mg/liter, respectively) (Tables 2 and S2). Omadacycline was as active (MIC_50_/MIC_90_ 2/4 mg/liter) against ceftazidime-NS (MIC, ≥8 mg/liter; AmpC-derepressed phenotype isolates) E. cloacae isolates as it was against ceftazidime-S (MIC_50_/MIC_90_, 2/4 mg/liter) E. cloacae isolates (Tables 2 and S2). Omadacycline was slightly less active against tetracycline-R isolates of the following organisms than it was against all isolates of the named organisms: E. coli (MIC_50_s/MIC_90_s, 1/4 mg/liter versus 0.5/2 mg/liter [all E. coli isolates]), K. pneumoniae (MIC_50_s/MIC_90_s, 4/16 mg/liter versus 2/8 mg/liter [all K. pneumoniae isolates]), Klebsiella oxytoca (MIC_50_s/MIC_90_s, 2/16 mg/liter versus 1/2 mg/liter [all K. oxytoca isolates]), E. cloacae SC (MIC_50_s/MIC_90_s, 4/16 mg/liter versus 2/4 mg/liter [all E. cloacae SC isolates]), and Citrobacter spp. (MIC_50_s/MIC_90_s, 4/8 mg/liter versus 1/4 mg/liter [all Citrobacter species isolates]) (Tables 2 and S2). Against A. baumannii, omadacycline (MIC_50_/MIC_90_, 4/8 mg/liter) inhibited 71.2% of isolates at ≤4 mg/liter (Table 2). Omadacycline inhibited 100.0% of other Acinetobacter species isolates (MIC_50_/MIC_90_, 0.25/0.5 mg/liter) at ≤4 mg/liter (data not shown). Omadacycline (MIC_50_/MIC_90_, 2/8 mg/liter; 82.2% of isolates were inhibited at ≤4 mg/liter) demonstrated good *in vitro* activity against S. maltophilia (Table 2). Omadacycline was equally active against β-lactamase-negative and -positive isolates of H. influenzae (MIC_50_/MIC_90_, 1/1 mg/liter) and was also very active against Moraxella catarrhalis isolates (MIC_50_/MIC_90_, 0.25/0.25 mg/liter) (Tables 2 and S2).

Compared to older tetracyclines, omadacycline has advantages that include a low propensity for selection of resistance, enhanced binding to the 30S ribosomal subunit, the ability to overcome common tetracycline resistance mechanisms, a lack of effect of other resistance mechanisms, availability as intravenous or oral formulations, a prolonged half-life, and once-daily administration (2). Omadacycline has recently completed phase 3 clinical trials for the treatment of ABSSSI and CABP.

This study documents the *in vitro* activity of omadacycline against bacterial isolates (United States and Europe) from the 2016 SENTRY survey. Overall, omadacycline provided broad coverage against Gram-positive and fastidious Gram-negative bacteria (Tables 1, 2, S1, and S2). The most active agents against staphylococci and streptococci were omadacycline, daptomycin, and linezolid. Omadacycline was active against MRSA, MR-CoNS, VRE, VGS, BHS, and penicillin- and macrolide-R S. pneumoniae isolates (Tables 1 and S1). Tetracycline-R Gram-positive strains remained susceptible to omadacycline. Omadacycline was active against ESBL-producing strains of E. coli and somewhat less active against ESBL-producing K. pneumoniae and ceftazidime-NS E. cloacae strains. Tetracycline-R Enterobacteriaceae were slightly less susceptible to omadacycline than tetracycline-S strains. Imipenem and amikacin were the most active agents against Enterobacteriaceae, including the resistant subsets. Omadacycline was the only agent with useful activity against A. baumannii, and omadacycline and trimethoprim-sulfamethoxazole were the only agents with useful activity against S. maltophilia isolates.

These data build on previous SENTRY surveillance surveys (12) and indicate that omadacycline is active against tetracycline-S and -R Gram-positive and -negative bacterial species and merits further study in the treatment of ABSSSI, CABP, and urinary tract infections.

## Supplementary Material

Supplemental material
