# Supplementary material for: Surveillance of Omadacycline Activity Tested against Clinical Isolates from the United States and Europe as Part of the 2016 SENTRY Antimicrobial Surveillance Program
Source: Antimicrob Agents Chemother. 2018 Mar 27;62(4):e02327-17. doi: 10.1128/AAC.02327-17 (PMC5913935; doi:10.1128/AAC.02327-17)
Supplement: Supplemental material [file supp_62_4_e02327-17__index.html]

Surveillance of Omadacycline Activity Tested against Clinical Isolates from the United States and Europe as Part of the 2016 SENTRY Antimicrobial Surveillance Program — Supplemental material 

# Surveillance of Omadacycline Activity Tested against Clinical Isolates from the United States and Europe as Part of the 2016 SENTRY Antimicrobial Surveillance Program

## Supplemental material

- Supplemental file 1 -

  Tables S1 and S2

  PDF, 800K
