# Supplementary material for: Surveillance of Omadacycline Activity Tested against Clinical Isolates from the United States and Europe as Part of the 2016 SENTRY Antimicrobial Surveillance Program
Source: Antimicrob Agents Chemother. 2018 Mar 27;62(4):e02327-17. doi: 10.1128/AAC.02327-17 (PMC5913935; doi:10.1128/AAC.02327-17)
Supplement: Supplemental material [file AAC.02327-17_zac004187036s1.pdf]

Table S1 Activity of omadacycline and comparator antimicrobial agents when tested against gram-positive isolates

Organism group (no. tested)<br>antimicrobial agent	CLSI[a]<br>%S	EUCAST[a]<br>%S	$MIC_{50/90}$ (mg/L)	MIC range (mg/L)
***Staphylococcus aureus* (4,215)**				
Omadacycline			0.12 / 0.25	≤0.015 – 8
Tetracycline	94.2	92.9	≤0.5 / ≤0.5	≤0.5 – >8
Tigecycline	>99.9	>99.9	0.06 / 0.12	≤0.015 – 1
Oxacillin	65.9	65.9	0.5 / >2	≤0.25 – >2
Levofloxacin	71.5	71.5	0.25 / >4	≤0.03 – >4
Erythromycin	58.2	58.7	0.25 / >8	≤0.06 – >8
Clindamycin	89.1	89.0	≤0.25 / >2	≤0.25 – >2
Linezolid	100.0	100.0	1 / 1	≤0.12 – 4
Daptomycin	100.0	100.0	0.5 / 0.5	≤0.12 – 1
Vancomycin	100.0	100.0	0.5 / 1	0.25 – 2
Gentamicin	96.2	95.8	≤1 / ≤1	≤1 – >8
Trimethoprim-sulfamethoxazole	98.5	98.5	≤0.5 / ≤0.5	≤0.5 – >4
**MSSA (2,777)**				
Omadacycline			0.12 / 0.25	≤0.015 – 1
Tetracycline	96.3	95.4	≤0.5 / ≤0.5	≤0.5 – >8
Tigecycline	100.0	100.0	0.06 / 0.12	≤0.015 – 0.25
Levofloxacin	93.6	93.6	0.25 / 0.5	≤0.03 – >4
Erythromycin	77.2	77.7	0.25 / >8	≤0.06 – >8

Organism group (no. tested) antimicrobial agent	CLSI[a] %S	EUCAST[a] %S	$MIC_{50/90}$ (mg/L)	MIC range (mg/L)
Clindamycin	97.8	97.6	≤0.25 / ≤0.25	≤0.25 – >2
Linezolid	100.0	100.0	1 / 2	≤0.12 – 2
Daptomycin	100.0	100.0	0.5 / 0.5	≤0.12 – 1
Vancomycin	100.0	100.0	0.5 / 1	0.25 – 2
Gentamicin	98.1	97.7	≤1 / ≤1	≤1 – >8
Trimethoprim-sulfamethoxazole	99.6	99.6	≤0.5 / ≤0.5	≤0.5 – >4
**MRSA (1,438)**				
Omadacycline			0.12 / 0.25	0.03 – 8
Tetracycline	90.3	88.0	≤0.5 / 4	≤0.5 – >8
Tigecycline	99.9	99.9	0.06 / 0.12	≤0.015 – 1
Levofloxacin	28.9	28.9	4 / >4	0.12 – >4
Erythromycin	21.6	22.1	>8 / >8	≤0.06 – >8
Clindamycin	72.3	72.3	≤0.25 / >2	≤0.25 – >2
Linezolid	100.0	100.0	1 / 1	0.25 – 4
Daptomycin	100.0	100.0	0.5 / 0.5	≤0.12 – 1
Vancomycin	100.0	100.0	0.5 / 1	0.25 – 2
Gentamicin	92.5	92.3	≤1 / ≤1	≤1 – >8
Trimethoprim-sulfamethoxazole	96.3	96.3	≤0.5 / ≤0.5	≤0.5 – >4
**Tetracycline-resistant *Staphylococcus aureus* (221)**				
Omadacycline			0.12 / 0.5	0.03 – 2
Tigecycline	99.5	99.5	0.12 / 0.25	0.03 – 1

Organism group (no. tested) antimicrobial agent	CLSI[a] %S	EUCAST[a] %S	$MIC_{50/90}$ (mg/L)	MIC range (mg/L)
Oxacillin	42.5	42.5	>2 / >2	≤0.25 – >2
Levofloxacin	68.8	68.8	0.25 / >4	0.06 – >4
Erythromycin	39.4	39.4	8 / >8	≤0.06 – >8
Clindamycin	72.9	72.9	≤0.25 / >2	≤0.25 – >2
Linezolid	100.0	100.0	1 / 1	0.25 – 2
Daptomycin	100.0	100.0	0.5 / 0.5	≤0.12 – 1
Vancomycin	100.0	100.0	1 / 1	0.5 – 2
Gentamicin	85.5	84.6	≤1 / >8	≤1 – >8
Trimethoprim-sulfamethoxazole	93.7	93.7	≤0.5 / 1	≤0.5 – >4
**Coagulase-negative staphylococci (723)**				
Omadacycline			0.12 / 0.5	≤0.015 – 2
Tetracycline	86.3	81.7	≤0.5 / >8	≤0.5 – >8
Tigecycline		100.0	0.06 / 0.12	≤0.015 – 0.5
Oxacillin	36.4	38.6	>2 / >2	≤0.25 – >2
Levofloxacin	55.4	55.4	0.5 / >4	≤0.03 – >4
Erythromycin	39.8	40.5	>8 / >8	≤0.06 – >8
Clindamycin	73.7	72.8	≤0.25 / >2	≤0.25 – >2
Linezolid	98.5	98.5	0.5 / 1	≤0.12 – >8
Daptomycin	99.9	99.9	0.5 / 0.5	≤0.12 – 2
Vancomycin	100.0	100.0	1 / 2	≤0.12 – 4
Gentamicin	68.9	65.3	≤1 / >8	≤1 – >8
Trimethoprim-sulfamethoxazole	69.2	69.2	≤0.5 / >4	≤0.5 – >4

Organism group (no. tested) antimicrobial agent	CLSI[a] %S	EUCAST[a] %S	$MIC_{50/90}$ (mg/L)	MIC range (mg/L)
**MS-CoNS (263)**				
Omadacycline			0.06 / 0.5	≤0.015 – 1
Tetracycline	94.7	92.8	≤0.5 / 1	≤0.5 – >8
Tigecycline		100.0	0.06 / 0.12	≤0.015 – 0.5
Levofloxacin	92.8	92.8	0.25 / 0.5	≤0.03 – >4
Erythromycin	70.7	70.7	0.12 / >8	≤0.06 – >8
Clindamycin	93.9	92.8	≤0.25 / ≤0.25	≤0.25 – >2
Linezolid	100.0	100.0	0.5 / 1	≤0.12 – 1
Daptomycin	100.0	100.0	0.25 / 0.5	≤0.12 – 1
Vancomycin	100.0	100.0	1 / 1	≤0.12 – 2
Gentamicin	96.6	95.8	≤1 / ≤1	≤1 – >8
Trimethoprim-sulfamethoxazole	92.4	92.4	≤0.5 / ≤0.5	≤0.5 – >4
**MR-CoNS (460)**				
Omadacycline			0.12 / 0.5	≤0.015 – 2
Tetracycline	81.5	75.4	≤0.5 / >8	≤0.5 – >8
Tigecycline		100.0	0.12 / 0.25	≤0.015 – 0.5
Levofloxacin	34.0	34.0	4 / >4	0.06 – >4
Erythromycin	22.2	23.3	>8 / >8	≤0.06 – >8
Clindamycin	62.2	61.3	≤0.25 / >2	≤0.25 – >2
Linezolid	97.6	97.6	0.5 / 1	≤0.12 – >8
Daptomycin	99.8	99.8	0.5 / 0.5	≤0.12 – 2

Organism group (no. tested)<br>antimicrobial agent	CLSI[a] %S	EUCAST[a] %S	$MIC_{50/90}$ (mg/L)	MIC range (mg/L)
Vancomycin	100.0	100.0	1 / 2	≤0.12 – 4
Gentamicin	53.0	47.8	2 / >8	≤1 – >8
Trimethoprim-sulfamethoxazole	55.9	55.9	2 / >4	≤0.5 – >4
**Tetracycline-resistant coagulase-negative staphylococci (92)**				
Omadacycline			0.25 / 0.5	0.03 – 2
Tigecycline		100.0	0.12 / 0.25	0.06 – 0.5
Oxacillin	14.1	14.1	>2 / >2	≤0.25 – >2
Levofloxacin	44.6	44.6	4 / >4	0.06 – >4
Erythromycin	22.8	25.0	>8 / >8	≤0.06 – >8
Clindamycin	69.6	69.6	≤0.25 / >2	≤0.25 – >2
Linezolid	95.7	95.7	1 / 1	0.25 – >8
Daptomycin	100.0	100.0	0.5 / 1	≤0.12 – 1
Vancomycin	100.0	100.0	1 / 2	0.25 – 2
Gentamicin	73.9	60.9	≤1 / >8	≤1 – >8
Trimethoprim-sulfamethoxazole	59.8	59.8	2 / >4	≤0.5 – >4
Enterococcus faecalis **(677)**				
Omadacycline			0.12 / 0.25	≤0.015 – 1
Tetracycline	21.4		>16 / >16	≤0.12 – >16
Tigecycline	100.0	100.0	0.06 / 0.12	≤0.015 – 0.12
Piperacillin-tazobactam		100.0	4 / 8	0.25 – >16
Levofloxacin	74.3	75.0[b]	1 / >4	≤0.03 – >4
Erythromycin	12.6		>16 / >16	≤0.12 – >16

Organism group (no. tested)<br>antimicrobial agent	CLSI[a] %S	EUCAST[a] %S	$MIC_{50/90}$ (mg/L)	MIC range (mg/L)
Linezolid	100.0	100.0	1 / 2	0.25 – 2
Daptomycin	100.0		1 / 1	≤0.25 – 4
Vancomycin	97.9	97.9	1 / 2	0.25 – >16
Ampicillin	100.0	100.0	1 / 2	≤0.5 – 4
**Vancomycin-susceptible *Enterococcus faecalis* (663)**				
Omadacycline			0.12 / 0.25	≤0.015 – 1
Tetracycline	21.7		>16 / >16	≤0.12 – >16
Tigecycline	100.0	100.0	0.06 / 0.12	≤0.015 – 0.12
Piperacillin-tazobactam		100.0	4 / 8	0.25 – >16
Levofloxacin	75.7	76.5[b]	1 / >4	≤0.03 – >4
Erythromycin	12.8		>16 / >16	≤0.12 – >16
Linezolid	100.0	100.0	1 / 2	0.25 – 2
Daptomycin	100.0		1 / 1	≤0.25 – 4
Ampicillin	100.0	100.0	1 / 2	≤0.5 – 4
**Vancomycin-nonsusceptible *Enterococcus faecalis* (14)**				
Omadacycline			0.12 / 0.25	≤0.015–0.25
Tetracycline	7.7		>16 / >16	0.25 – >16
Tigecycline	100.0	100.0	0.06 / 0.12	≤0.015 – 0.12
Piperacillin-tazobactam		100.0	8 / 16	2 – 16
Levofloxacin	7.1	7.1[b]	>4 / >4	1 – >4
Erythromycin	0.0		>16 / >16	2 – >16
Linezolid	100.0	100.0	1 / 1	0.5 – 2

Organism group (no. tested)<br>antimicrobial agent	CLSI[a]<br>%S	EUCAST[a]<br>%S	$MIC_{50/90}$ (mg/L)	MIC range (mg/L)
Daptomycin	100.0		0.5 / 1	0.5 – 1
Ampicillin	100.0	100.0	1 / 2	≤0.5 – 2
**Tetracycline-resistant *Enterococcus faecalis* (524)**				
Omadacycline			0.12 / 0.25	≤0.015 – 1
Tigecycline	100.0	100.0	0.06 / 0.12	≤0.015 – 0.12
Piperacillin-tazobactam		100.0	4 / 8	0.25 – >16
Levofloxacin	70.0	71.0[b]	1 / >4	≤0.03 – >4
Erythromycin	10.1		>16 / >16	≤0.12 – >16
Linezolid	100.0	100.0	1 / 2	0.25 – 2
Daptomycin	100.0		1 / 1	≤0.25 – 4
Vancomycin	97.9	97.9	1 / 2	0.25 – >16
Ampicillin	100.0	100.0	1 / 2	≤0.5 – 4
***Enterococcus faecium* (390)**				
Omadacycline			0.06 / 0.12	≤0.015 – 8
Tetracycline	42.6		16 / >16	≤0.12 – >16
Tigecycline		99.5	0.03 / 0.06	≤0.015 – 1
Piperacillin-tazobactam		12.9	>16 / >16	0.5 – >16
Levofloxacin	9.0	13.1[b]	>4 / >4	0.5 – >4
Erythromycin	5.6		>16 / >16	≤0.12 – >16
Linezolid	99.5	99.5	1 / 2	0.25 – 8
Daptomycin	99.7		1 / 2	≤0.25 – 8
Vancomycin	60.0	60.0	1 / >16	0.25 – >16

Organism group (no. tested)<br>antimicrobial agent	CLSI[a] %S	EUCAST[a] %S	$MIC_{50/90}$ (mg/L)	MIC range (mg/L)
Ampicillin	14.1	13.1	>16 / >16	≤0.5 – >16
**Vancomycin-susceptible *Enterococcus faecium* (234)**				
Omadacycline			0.06 / 0.12	≤0.015 – 1
Tetracycline	58.1		0.5 / >16	≤0.12 – >16
Tigecycline		100.0	0.03 / 0.06	≤0.015 – 0.25
Piperacillin-tazobactam		20.2	>16 / >16	0.5 – >16
Levofloxacin	15.0	20.9[b]	>4 / >4	0.5 – >4
Erythromycin	7.7		>16 / >16	≤0.12 – >16
Linezolid	99.6	99.6	1 / 2	0.25 – 8
Daptomycin	100.0		2 / 2	≤0.25 – 4
Ampicillin	21.8	20.5	>16 / >16	≤0.5 – >16
**Vancomycin-nonsusceptible *Enterococcus faecium* (156)**				
Omadacycline			0.06 / 0.12	≤0.015 – 8
Tetracycline	19.2		>16 / >16	≤0.12 – >16
Tigecycline		98.7	0.03 / 0.06	≤0.015 – 1
Piperacillin-tazobactam		1.9	>16 / >16	2 – >16
Levofloxacin	0.0	1.3[b]	>4 / >4	4 – >4
Erythromycin	2.6		>16 / >16	≤0.12 – >16
Linezolid	99.4	99.4	1 / 1	0.25 – 8
Daptomycin	99.4		1 / 2	≤0.25 – 8
Ampicillin	2.6	1.9	>16 / >16	≤0.5 – >16
**Tetracycline-resistant *Enterococcus faecium* (217)**				

Organism group (no. tested) antimicrobial agent	CLSI[a] %S	EUCAST[a] %S	$MIC_{50/90}$ (mg/L)	MIC range (mg/L)
Omadacycline			0.12 / 0.12	≤0.015 – 8
Tigecycline		99.1	0.06 / 0.06	≤0.015 – 1
Piperacillin-tazobactam		6.0	>16 / >16	2 – >16
Levofloxacin	4.1	8.8[b]	>4 / >4	0.5 – >4
Erythromycin	4.1		>16 / >16	≤0.12 – >16
Linezolid	99.5	99.5	1 / 1	0.25 – 8
Daptomycin	99.5		1 / 2	≤0.25 – 8
Vancomycin	44.7	44.7	>16 / >16	0.25 – >16
Ampicillin	6.9	6.0	>16 / >16	≤0.5 – >16
***Streptococcus pneumoniae* (1,314)**				
Omadacycline			0.06 / 0.12	≤0.015 – 1
Tetracycline	79.5	79.5	≤0.25 / >8	≤0.25 – >8
Tigecycline	99.4		0.03 / 0.06	0.015 – 0.25
Ceftriaxone	87.1[c] 96.9[d]	87.1	0.03 / 1	≤0.015 – >2
Amoxicillin-clavulanic acid	95.0		≤0.03 / 2	≤0.03 – >4
Levofloxacin	98.6	98.6	1 / 1	0.25 – >4
Erythromycin	67.0	67.0	0.06 / >32	≤0.015 – >32
Clindamycin	84.9	85.2	≤0.25 / >2	≤0.25 – >2
Linezolid	100.0	100.0	1 / 2	0.25 – 2
Vancomycin	100.0	100.0	0.25 / 0.5	≤0.06 – 0.5
Azithromycin	67.0	66.4	0.06 / >32	0.008 – >32

Organism group (no. tested)<br>antimicrobial agent	CLSI[a] %S	EUCAST[a] %S	$MIC_{50/90}$ (mg/L)	MIC range (mg/L)
Penicillin	68.4[e]<br>68.4[f]<br>95.7[g]	68.4[c]<br>68.4[d]	0.015 / 2	≤0.004 – >8
Trimethoprim-sulfamethoxazole	72.8	78.7	0.25 / >4	≤0.12 – >4
**Penicillin-susceptible *Streptococcus pneumoniae* (899)**				
Omadacycline			0.06 / 0.06	≤0.015 – 0.5
Tetracycline	92.2	92.2	≤0.25 / 0.5	≤0.25 – >8
Tigecycline	99.4		0.03 / 0.06	0.015 – 0.12
Ceftriaxone	100.0[c]<br>100.0[d]	100.0	0.03 / 0.06	≤0.015 – 0.5
Amoxicillin-clavulanic acid	100.0		≤0.03 / ≤0.03	≤0.03 – 0.12
Levofloxacin	98.4	98.4	1 / 2	0.25 – >4
Erythromycin	83.9	83.9	0.03 / 8	≤0.015 – >32
Clindamycin	95.4	95.6	≤0.25 / ≤0.25	≤0.25 – >2
Linezolid	100.0	100.0	1 / 2	0.25 – 2
Vancomycin	100.0	100.0	0.25 / 0.25	≤0.06 – 0.5
Azithromycin	84.1	83.8	0.06 / 8	0.008 – >32
Trimethoprim-sulfamethoxazole	87.5	91.8	0.25 / 1	≤0.12 – >4
**Penicillin-intermediate *Streptococcus pneumoniae* (263)**				
Omadacycline			0.06 / 0.12	≤0.015 – 1
Tetracycline	59.7	59.7	0.5 / >8	≤0.25 – >8

Organism group (no. tested) antimicrobial agent	CLSI[a] %S	EUCAST[a] %S	$MIC_{50/90}$ (mg/L)	MIC range (mg/L)
Tigecycline	98.9		0.03 / 0.06	0.015 – 0.25
Ceftriaxone	88.6[c] 100.0[d]	88.6	0.25 / 1	≤0.015 – 1
Amoxicillin-clavulanic acid	99.2		0.25 / 2	≤0.03 – 4
Levofloxacin	98.9	98.9	1 / 1	0.25 – >4
Erythromycin	38.0	38.0	4 / >32	≤0.015 – >32
Clindamycin	70.7	71.1	≤0.25 / >2	≤0.25 – >2
Linezolid	100.0	100.0	1 / 2	0.25 – 2
Vancomycin	100.0	100.0	0.25 / 0.25	≤0.06 – 0.5
Azithromycin	37.3	35.4	8 / >32	0.008 – >32
Trimethoprim-sulfamethoxazole	47.1	59.3	1 / >4	≤0.12 – >4
**Penicillin-resistant *Streptococcus pneumoniae* (152)**				
Omadacycline			0.06 / 0.12	≤0.015 – 0.12
Tetracycline	38.8	38.8	>8 / >8	≤0.25 – >8
Tigecycline	100.0		0.06 / 0.06	0.015 – 0.06
Ceftriaxone	7.9[c] 73.0[d]	7.9	1 / 2	0.5 – >2
Amoxicillin-clavulanic acid	57.9		2 / >4	0.5 – >4
Levofloxacin	98.7	98.7	1 / 1	0.5 – >4
Erythromycin	17.8	17.8	>32 / >32	0.03 – >32
Clindamycin	46.7	48.0	>2 / >2	≤0.25 – >2
Linezolid	100.0	100.0	1 / 1	0.25 – 2
Vancomycin	100.0	100.0	0.25 / 0.5	0.12 – 0.5

Organism group (no. tested)<br>antimicrobial agent	CLSI[a] %S	EUCAST[a] %S	$MIC_{50/90}$ (mg/L)	MIC range (mg/L)
Azithromycin	17.8	17.8	>32 / >32	0.03 – >32
Trimethoprim-sulfamethoxazole	30.3	34.9	4 / >4	≤0.12 – >4
**Macrolide-resistant (erythromycin and azithromycin) *Streptococcus pneumoniae* (413)**				
Omadacycline			0.06 / 0.12	≤0.015 – 1
Tetracycline	44.1	44.1	>8 / >8	≤0.25 – >8
Tigecycline	99.5		0.06 / 0.06	0.015 – 0.25
Ceftriaxone	66.3[c]<br>91.3[d]	66.3	0.25 / 1	≤0.015 – >2
Amoxicillin-clavulanic acid	87.2		0.5 / 4	≤0.03 – >4
Levofloxacin	98.8	98.8	1 / 1	0.25 – >4
Clindamycin	52.3	53.0	≤0.25 / >2	≤0.25 – >2
Linezolid	100.0	100.0	1 / 2	0.25 – 2
Vancomycin	100.0	100.0	0.25 / 0.5	≤0.06 – 0.5
Penicillin	32.4[e]<br>32.4[f]<br>89.3[g]	32.4[c]<br>32.4[d]	0.25 / 4	0.008 – >8
Trimethoprim-sulfamethoxazole	52.5	61.5	0.5 / >4	≤0.12 – >4
**Tetracycline-resistant *Streptococcus pneumoniae* (263)**				
Omadacycline			0.06 / 0.12	≤0.015 – 1
Tigecycline	99.2		0.06 / 0.06	0.015 – 0.25
Ceftriaxone	61.2[c]<br>87.5[d]	61.2	0.25 / 2	≤0.015 – >2

Organism group (no. tested) antimicrobial agent	CLSI[a] %S	EUCAST[a] %S	$MIC_{50/90}$ (mg/L)	MIC range (mg/L)
Amoxicillin-clavulanic acid	81.0		0.5 / 4	≤0.03 – >4
Levofloxacin	97.3	97.3	1 / 1	0.25 – >4
Erythromycin	10.6	10.6	>32 / >32	≤0.015 – >32
Clindamycin	32.7	33.8	>2 / >2	≤0.25 – >2
Linezolid	100.0	100.0	1 / 1	0.25 – 2
Vancomycin	100.0	100.0	0.25 / 0.5	≤0.06 – 0.5
Azithromycin	10.6	10.3	>32 / >32	0.03 – >32
Penicillin	25.5[e]<br>25.5[f]<br>83.7[g]	25.5[c]<br>25.5[d]	0.5 / 4	0.008 – >8
Trimethoprim-sulfamethoxazole	38.4	51.3	1 / >4	≤0.12 – >4
**Viridans group streptococci (327)**				
Omadacycline			0.06 / 0.12	≤0.015 – 0.5
Tetracycline	62.7		0.5 / >8	≤0.25 – >8
Tigecycline	100.0		0.03 / 0.06	≤0.008 – 0.25
Ceftriaxone	90.2	85.9	0.12 / 1	≤0.015 – >2
Amoxicillin-clavulanic acid		79.8	0.06 / 2	≤0.03 – >4
Piperacillin-tazobactam		79.8	0.25 / 4	≤0.06 – >8
Levofloxacin	94.2		1 / 2	0.25 – >4
Erythromycin	55.0		0.03 / >32	≤0.015 – >32
Clindamycin	86.2	87.8	≤0.25 / >2	≤0.25 – >2
Linezolid	99.7		1 / 1	0.12 – >4
Daptomycin	100.0		0.25 / 1	≤0.06 – 1

Organism group (no. tested)<br>antimicrobial agent	CLSI[a] %S	EUCAST[a] %S	$MIC_{50/90}$ (mg/L)	MIC range (mg/L)
Vancomycin	100.0	100.0	0.5 / 0.5	≤0.06 – 1
Penicillin	70.9	79.8	0.06 / 2	≤0.004 – >8
**Tetracycline-resistant viridans group streptococci (112)**				
Omadacycline			0.12 / 0.25	≤0.015 – 0.5
Tigecycline	100.0		0.06 / 0.12	0.015 – 0.25
Ceftriaxone	87.5	83.0	0.12 / 2	≤0.015 – >2
Amoxicillin-clavulanic acid		74.1	0.06 / 2	≤0.03 – >4
Piperacillin-tazobactam		74.1	0.25 / 4	≤0.06 – >8
Levofloxacin	92.0		1 / 2	0.25 – >4
Erythromycin	39.3		2 / >32	≤0.015 – >32
Clindamycin	67.0	71.4	≤0.25 / >2	≤0.25 – >2
Linezolid	99.1		1 / 1	0.12 – >4
Daptomycin	100.0		0.25 / 0.5	≤0.06 – 1
Vancomycin	100.0	100.0	0.5 / 0.5	≤0.06 – 1
Penicillin	64.3	74.1	0.06 / 2	≤0.004 – >8
***Streptococcus anginosus* group (107)**				
Omadacycline			0.06 / 0.12	≤0.015 – 0.12
Tetracycline	67.3		0.5 / >8	≤0.25 – >8
Tigecycline	100.0		0.03 / 0.03	≤0.008 – 0.12
Ceftriaxone	99.1	99.1	0.25 / 0.25	≤0.015 – >2
Amoxicillin-clavulanic acid		98.1	0.06 / 0.12	≤0.03 – >4
Piperacillin-tazobactam		98.1	0.12 / 0.25	≤0.06 – 2

Organism group (no. tested) antimicrobial agent	CLSI[a] %S	EUCAST[a] %S	$MIC_{50/90}$ (mg/L)	MIC range (mg/L)
Levofloxacin	98.1		0.5 / 1	0.25 – >4
Erythromycin	81.3		≤0.015 / 4	≤0.015 – >32
Clindamycin	86.0	87.9	≤0.25 / >2	≤0.25 – >2
Linezolid	100.0		1 / 1	0.12 – 2
Daptomycin	100.0		0.25 / 0.5	≤0.06 – 1
Vancomycin	100.0	100.0	0.5 / 1	≤0.06 – 1
Penicillin	98.1	98.1	0.03 / 0.06	≤0.004 – 4
**Tetracycline-resistant *Streptococcus anginosus* group (34)**				
Omadacycline			0.06 / 0.12	≤0.015 – 0.12
Tigecycline	100.0		0.06 / 0.12	≤0.015 – 0.12
Ceftriaxone	97.1	97.1	0.12 / 0.25	≤0.015 – >2
Amoxicillin-clavulanic acid		94.1	0.06 / 0.12	≤0.03 – >4
Piperacillin-tazobactam		94.1	≤0.06 / 0.25	≤0.06 – 2
Levofloxacin	94.1		0.5 / 1	0.25 – >4
Erythromycin	58.8		0.03 / >32	≤0.015 – >32
Clindamycin	67.6	73.5	≤0.25 / >2	≤0.25 – >2
Linezolid	100.0		1 / 1	0.5 – 2
Daptomycin	100.0		0.25 / 0.5	0.12 – 0.5
Vancomycin	100.0	100.0	0.5 / 1	≤0.06 – 1
Penicillin	94.1	94.1	0.03 / 0.06	≤0.004 – 4
**β-hemolytic streptococci (966)**				
Omadacycline			0.06 / 0.12	0.03 – 0.5

Organism group (no. tested)<br>antimicrobial agent	CLSI[a] %S	EUCAST[a] %S	$MIC_{50/90}$ (mg/L)	MIC range (mg/L)
Tetracycline	54.7	53.9	0.5 / >8	≤0.25 – >8
Tigecycline	100.0	100.0	0.06 / 0.06	0.015 – 0.25
Levofloxacin	99.3	99.3	0.5 / 1	0.12 – >4
Erythromycin	71.5	71.5	0.03 / >32	≤0.015 – >32
Clindamycin	84.8	85.4	≤0.25 / >2	≤0.25 – >2
Linezolid	100.0	100.0	1 / 1	0.25 – 2
Daptomycin	100.0	100.0	≤0.06 / 0.25	≤0.06 – 1
Vancomycin	100.0	100.0	0.25 / 0.5	0.12 – 1
Penicillin	100.0	100.0	0.015 / 0.06	≤0.004 – 0.12
**Tetracycline-resistant β-hemolytic streptococci (421)**				
Omadacycline			0.12 / 0.25	0.03 – 0.5
Tigecycline	100.0	100.0	0.06 / 0.06	0.015 – 0.25
Levofloxacin	99.5	99.5	0.5 / 1	0.25 – 4
Erythromycin	52.7	52.7	0.06 / >32	≤0.015 – >32
Clindamycin	73.2	74.6	≤0.25 / >2	≤0.25 – >2
Linezolid	100.0	100.0	1 / 1	0.5 – 2
Daptomycin	100.0	100.0	0.25 / 0.25	≤0.06 – 0.5
Vancomycin	100.0	100.0	0.25 / 0.5	0.12 – 0.5
Penicillin	100.0	100.0	0.03 / 0.06	≤0.004 – 0.12
**Macrolide (erythromycin)-resistant β-hemolytic streptococci (266)**				
Omadacycline			0.12 / 0.25	0.03 – 0.5
Tetracycline	25.6	24.8	>8 / >8	≤0.25 – >8

Organism group (no. tested) antimicrobial agent	CLSI[a] %S	EUCAST[a] %S	$MIC_{50/90}$ (mg/L)	MIC range (mg/L)
Tigecycline	100.0	100.0	0.06 / 0.06	0.015 – 0.25
Levofloxacin	98.5	98.5	0.5 / 1	0.25 – >4
Clindamycin	47.4	48.1	>2 / >2	≤0.25 – >2
Linezolid	100.0	100.0	1 / 1	0.25 – 2
Daptomycin	100.0	100.0	0.25 / 0.25	≤0.06 – 0.5
Vancomycin	100.0	100.0	0.25 / 0.5	0.12 – 0.5
Penicillin	100.0	100.0	0.03 / 0.06	≤0.004 – 0.12

[a] Criteria as published by CLSI [2017] and EUCAST [2017]

[b] Uncomplicated UTI only

[c] Using meningitis breakpoints

[d] Using non meningitis breakpoints

[e] Using oral breakpoints

[f] Using parenteral, meningitis breakpoints

[g] Using parenteral, non-meningitis breakpoints

Table S2 Activity of omadacycline and comparator antimicrobial agents when tested against gram-negative isolates

Organism group (no. tested)<br>antimicrobial agent	CLSI[a]<br>%S	EUCAST[a]<br>%S	$MIC_{50/90}$ (mg/L)	MIC range (mg/L)
***Enterobacteriaceae* (8,345)**				
Omadacycline			1 / 8	0.12 – >32
Tetracycline	64.2		2 / >16	≤0.25 – >16
Tigecycline	97.8	92.9	0.25 / 1	≤0.06 – 8
Ceftriaxone	78.0	78.0	≤0.06 / >8	≤0.06 – >8
Piperacillin-tazobactam	88.4	84.4	2 / 32	≤0.5 – >64
Levofloxacin	79.5	75.3	0.06 / >4	≤0.03 – >4
Gentamicin	88.7	88.2	0.5 / >8	≤0.06 – >8
Amikacin	98.5	96.9	2 / 4	≤0.25 – >32
Aztreonam	80.9	78.8	0.12 / >16	≤0.03 – >16
Cefepime	84.3	82.6	≤0.12 / >16	≤0.12 – >16
Ceftazidime	82.8	79.0	0.25 / 32	≤0.015 – >32
Imipenem	91.2	96.7	≤0.12 / 1	≤0.12 – >8
Trimethoprim-sulfamethoxazole	73.4	73.4	≤0.5 / >4	≤0.5 – >4
**Ceftazidime-nonsusceptible *Enterobacteriaceae* (1,439)**				
Omadacycline			2 / 8	0.12 – >32
Tetracycline	44.5		16 / >16	0.5 – >16
Tigecycline	98.2	92.7	0.25 / 1	≤0.06 – 8
Ceftriaxone	1.7	1.7	>8 / >8	≤0.06 – >8
Piperacillin-tazobactam	50.7	38.2	16 / >64	≤0.5 – >64

Organism group (no. tested) antimicrobial agent	CLSI[a] %S	EUCAST[a] %S	MIC$_{50/90}$ (mg/L)	MIC range (mg/L)
Levofloxacin	39.6	32.5	>4 / >4	≤0.03 – >4
Gentamicin	58.3	57.3	1 / >8	≤0.06 – >8
Amikacin	92.0	85.6	2 / 16	≤0.25 – >32
Aztreonam	4.2	1.6	>16 / >16	0.25 – >16
Cefepime	25.1	19.1	>16 / >16	≤0.12 – >16
Imipenem	83.2	87.4	≤0.12 / 8	≤0.12 – >8
Trimethoprim-sulfamethoxazole	38.4	38.4	>4 / >4	≤0.5 – >4
**Tetracycline-resistant *Enterobacteriaceae* (2,737)**				
Omadacycline			2 / 16	0.12 – >32
Tigecycline	93.4	81.0	0.5 / 2	≤0.06 – 8
Ceftriaxone	65.8	65.8	0.12 / >8	≤0.06 – >8
Piperacillin-tazobactam	84.5	77.6	2 / 64	≤0.5 – >64
Levofloxacin	60.5	52.9	0.5 / >4	≤0.03 – >4
Gentamicin	76.4	75.6	1 / >8	≤0.06 – >8
Amikacin	98.0	95.5	2 / 8	≤0.25 – >32
Aztreonam	68.3	64.9	0.12 / >16	≤0.03 – >16
Cefepime	71.4	68.8	≤0.12 / >16	≤0.12 – >16
Ceftazidime	73.0	66.6	0.25 / >32	0.03 – >32
Imipenem	82.5	94.6	≤0.12 / 2	≤0.12 – >8
Trimethoprim-sulfamethoxazole	44.3	44.3	>4 / >4	≤0.5 – >4
***Escherichia coli* (3,541)**				
Omadacycline			0.5 / 2	0.12 – 32

Organism group (no. tested) antimicrobial agent	CLSI[a] %S	EUCAST[a] %S	$MIC_{50/90}$ (mg/L)	MIC range (mg/L)
Tetracycline	63.7		2 / >16	≤0.25 – >16
Tigecycline	99.9	99.9	0.12 / 0.25	≤0.06 – 4
Ceftriaxone	80.3	80.3	≤0.06 / >8	≤0.06 – >8
Piperacillin-tazobactam	93.3	89.7	2 / 16	≤0.5 – >64
Levofloxacin	69.6	68.1	≤0.03 / >4	≤0.03 – >4
Gentamicin	87.7	87.4	0.5 / >8	≤0.06 – >8
Amikacin	99.4	97.4	2 / 4	≤0.25 – >32
Aztreonam	82.7	80.0	0.12 / >16	≤0.03 – >16
Cefepime	82.6	81.2	≤0.12 / >16	≤0.12 – >16
Ceftazidime	85.6	80.8	0.25 / 16	0.03 – >32
Imipenem	99.8	99.9	≤0.12 / ≤0.12	≤0.12 – >8
Trimethoprim-sulfamethoxazole	64.1	64.1	≤0.5 / >4	≤0.5 – >4
**Ceftazidime-susceptible *Escherichia coli* (3,030)**				
Omadacycline			0.5 / 2	0.12 – 32
Tetracycline	69.1		2 / >16	≤0.25 – >16
Tigecycline	100.0	>99.9	0.12 / 0.25	≤0.06 – 2
Ceftriaxone	93.6	93.6	≤0.06 / 0.12	≤0.06 – >8
Piperacillin-tazobactam	95.9	94.4	2 / 4	≤0.5 – >64
Levofloxacin	78.4	77.1	≤0.03 / >4	≤0.03 – >4
Gentamicin	92.7	92.4	0.5 / 2	≤0.06 – >8
Amikacin	99.8	98.7	2 / 4	≤0.25 – >32
Aztreonam	96.1	93.3	0.06 / 0.25	≤0.03 – >16

Organism group (no. tested) antimicrobial agent	CLSI[a] %S	EUCAST[a] %S	$MIC_{50/90}$ (mg/L)	MIC range (mg/L)
Cefepime	94.8	93.5	≤0.12 / 0.25	≤0.12 – >16
Imipenem	99.9	100.0	≤0.12 / ≤0.12	≤0.12 – 2
Trimethoprim-sulfamethoxazole	68.6	68.6	≤0.5 / >4	≤0.5 – >4
**Ceftazidime-nonsusceptible *Escherichia coli* (511)**				
Omadacycline			1 / 2	0.12 – 32
Tetracycline	31.7		>16 / >16	0.5 – >16
Tigecycline	99.6	99.6	0.12 / 0.25	≤0.06 – 4
Ceftriaxone	1.4	1.4	>8 / >8	0.25 – >8
Piperacillin-tazobactam	78.0	62.2	8 / >64	≤0.5 – >64
Levofloxacin	17.4	14.9	>4 / >4	≤0.03 – >4
Gentamicin	57.7	57.7	1 / >8	0.25 – >8
Amikacin	97.1	89.8	4 / 16	0.5 – >32
Aztreonam	3.1	1.0	>16 / >16	1 – >16
Cefepime	10.2	8.2	>16 / >16	≤0.12 – >16
Imipenem	98.8	99.2	≤0.12 / 0.25	≤0.12 – >8
Trimethoprim-sulfamethoxazole	37.4	37.4	>4 / >4	≤0.5 – >4
**Tetracycline-resistant *Escherichia coli* (1,272)**				
Omadacycline			1 / 4	0.12 – 32
Tigecycline	99.8	99.8	0.12 / 0.25	≤0.06 – 4
Ceftriaxone	63.7	63.7	≤0.06 / >8	≤0.06 – >8
Piperacillin-tazobactam	88.5	80.6	2 / 32	≤0.5 – >64
Levofloxacin	46.0	43.7	4 / >4	≤0.03 – >4

Organism group (no. tested) antimicrobial agent	CLSI[a] %S	EUCAST[a] %S	$MIC_{50/90}$ (mg/L)	MIC range (mg/L)
Gentamicin	75.5	75.4	1 / >8	≤0.06 – >8
Amikacin	98.7	95.0	2 / 8	≤0.25 – >32
Aztreonam	67.7	63.4	0.12 / >16	≤0.03 – >16
Cefepime	67.4	64.9	≤0.12 / >16	≤0.12 – >16
Ceftazidime	72.7	64.9	0.25 / 32	0.03 – >32
Imipenem	99.6	99.8	≤0.12 / ≤0.12	≤0.12 – >8
Trimethoprim-sulfamethoxazole	29.5	29.5	>4 / >4	≤0.5 – >4
***Klebsiella pneumoniae* (1,771)**				
Omadacycline			2 / 8	0.25 – >32
Tetracycline	71.6		2 / >16	≤0.25 – >16
Tigecycline	98.8	94.5	0.25 / 1	≤0.06 – 4
Ceftriaxone	70.4	70.4	≤0.06 / >8	≤0.06 – >8
Piperacillin-tazobactam	78.4	72.4	4 / >64	≤0.5 – >64
Levofloxacin	78.2	72.7	0.06 / >4	≤0.03 – >4
Gentamicin	82.6	82.3	0.25 / >8	≤0.06 – >8
Amikacin	95.0	92.3	1 / 8	≤0.25 – >32
Aztreonam	71.7	70.4	0.06 / >16	≤0.03 – >16
Cefepime	72.2	71.3	≤0.12 / >16	≤0.12 – >16
Ceftazidime	71.4	69.7	0.25 / >32	0.03 – >32
Imipenem	90.3	91.9	≤0.12 / 1	≤0.12 – >8
Trimethoprim-sulfamethoxazole	69.7	69.7	≤0.5 / >4	≤0.5 – >4
**Ceftazidime-susceptible *Klebsiella pneumoniae* (1,264)**				

Organism group (no. tested) antimicrobial agent	CLSI[a] %S	EUCAST[a] %S	$MIC_{50/90}$ (mg/L)	MIC range (mg/L)
Omadacycline			1 / 4	0.25 – >32
Tetracycline	84.1		1 / >16	≤0.25 – >16
Tigecycline	99.3	96.6	0.25 / 0.5	≤0.06 – 4
Ceftriaxone	97.9	97.9	≤0.06 / 0.12	≤0.06 – >8
Piperacillin-tazobactam	95.8	91.5	2 / 8	≤0.5 – >64
Levofloxacin	97.0	93.1	0.06 / 0.5	≤0.03 – >4
Gentamicin	98.0	97.9	0.25 / 0.5	≤0.06 – >8
Amikacin	99.8	99.8	1 / 2	≤0.25 – >32
Aztreonam	99.3	98.3	0.06 / 0.12	≤0.03 – >16
Cefepime	98.6	98.0	≤0.12 / 0.25	≤0.12 – >16
Imipenem	99.6	100.0	≤0.12 / 0.25	≤0.12 – 2
Trimethoprim-sulfamethoxazole	90.8	90.8	≤0.5 / 2	≤0.5 – >4
**Ceftazidime-nonsusceptible *Klebsiella pneumoniae* (507)**				
Omadacycline			2 / 8	0.25 – >32
Tetracycline	40.4		16 / >16	0.5 – >16
Tigecycline	97.4	89.3	0.5 / 2	≤0.06 – 4
Ceftriaxone	1.6	1.6	>8 / >8	0.25 – >8
Piperacillin-tazobactam	34.9	24.9	64 / >64	1 – >64
Levofloxacin	31.4	21.7	>4 / >4	≤0.03 – >4
Gentamicin	44.2	43.4	>8 / >8	0.12 – >8
Amikacin	83.2	73.8	4 / 32	0.5 – >32
Aztreonam	3.0	0.8	>16 / >16	0.25 – >16

Organism group (no. tested) antimicrobial agent	CLSI[a] %S	EUCAST[a] %S	$MIC_{50/90}$ (mg/L)	MIC range (mg/L)
Cefepime	6.3	4.7	>16 / >16	≤0.12 – >16
Imipenem	67.3	71.6	0.25 / >8	≤0.12 – >8
Trimethoprim-sulfamethoxazole	17.0	17.0	>4 / >4	≤0.5 – >4
**Tetracycline-resistant *Klebsiella pneumoniae* (430)**				
Omadacycline			4 / 16	0.5 – >32
Tigecycline	94.9	82.6	0.5 / 2	≤0.06 – 4
Ceftriaxone	36.7	36.7	>8 / >8	≤0.06 – >8
Piperacillin-tazobactam	54.4	41.4	16 / >64	1 – >64
Levofloxacin	54.8	44.5	1 / >4	≤0.03 – >4
Gentamicin	57.9	57.2	0.5 / >8	0.12 – >8
Amikacin	94.2	90.5	2 / 8	0.5 – >32
Aztreonam	39.3	36.5	>16 / >16	≤0.03 – >16
Cefepime	39.0	36.7	>16 / >16	≤0.12 – >16
Ceftazidime	38.4	34.9	16 / >32	0.06 – >32
Imipenem	84.4	88.1	≤0.12 / 4	≤0.12 – >8
Trimethoprim-sulfamethoxazole	25.8	25.8	>4 / >4	≤0.5 – >4
**Tigecycline-nonsusceptible *Klebsiella pneumoniae* (22)**				
Omadacycline			32 / >32	16 - >32
Tetracycline	0.0		>16 / >16	16 - >16
Ceftriaxone	45.5	45.5	>8 / >8	0.12 - >8
Piperacillin-tazobactam	45.5	9.1	32 / >64	4 - >64
Levofloxacin	45.5	36.4	4 / >4	0.5 - >4

Organism group (no. tested) antimicrobial agent	CLSI[a] %S	EUCAST[a] %S	$MIC_{50/90}$ (mg/L)	MIC range (mg/L)
Gentamicin	59.1	59.1	0.25 / >8	0.12 - >8
Amikacin	95.5	86.4	1 / 16	0.5 - >32
Aztreonam	45.5	40.9	>16 / >16	0.12 - >16
Cefepime	45.5	40.9	>16 / >16	0.25 - >16
Ceftazidime	40.9	36.4	32 / >32	0.5 - >32
Imipenem	90.9	95.5	≤0.12 / 1	≤0.12 - >8
Trimethoprim-sulfamethoxazole	22.7	22.7	>4 / >4	≤0.5 - >4
***Klebsiella oxytoca* (423)**				
Omadacycline			1 / 2	0.25 – 32
Tetracycline	89.8		1 / 8	≤0.25 – >16
Tigecycline	100.0	97.6	0.25 / 0.5	≤0.06 – 2
Ceftriaxone	85.3	85.3	≤0.06 / >8	≤0.06 – >8
Piperacillin-tazobactam	85.8	82.7	2 / >64	≤0.5 – >64
Levofloxacin	96.0	92.0	≤0.03 / 0.25	≤0.03 – >4
Gentamicin	96.4	96.0	0.5 / 1	≤0.06 – >8
Amikacin	100.0	99.8	1 / 2	≤0.25 – 16
Aztreonam	85.8	83.9	0.25 / >16	≤0.03 – >16
Cefepime	92.4	90.3	≤0.12 / 1	≤0.12 – >16
Ceftazidime	95.0	91.3	0.12 / 1	0.03 – >32
Imipenem	98.1	98.1	≤0.12 / 0.25	≤0.12 – >8
Trimethoprim-sulfamethoxazole	89.8	89.8	≤0.5 / 4	≤0.5 – >4
**Tetracycline-resistant *Klebsiella oxytoca* (30)**				

Organism group (no. tested) antimicrobial agent	CLSI[a] %S	EUCAST[a] %S	$MIC_{50/90}$ (mg/L)	MIC range (mg/L)
Omadacycline			2 / 16	0.25 – 32
Tigecycline	100.0	90.0	0.5 / 1	≤0.06 / 2
Ceftriaxone	50.0	50.0	0.5 / >8	≤0.06 – >8
Piperacillin-tazobactam	53.3	46.7	16 / >64	≤0.5 – >64
Levofloxacin	70.0	50.0	0.5 / >4	≤0.03 – >4
Gentamicin	80.0	80.0	0.5 / >8	0.25 – >8
Amikacin	100.0	100.0	2 / 4	0.5 – 8
Aztreonam	50.0	50.0	0.5 / >16	0.06 – >16
Cefepime	56.7	53.3	1 / 16	≤0.12 – >16
Ceftazidime	83.3	60.0	1 / 16	0.06 – >32
Imipenem	96.7	96.7	≤0.12 / ≤0.12	≤0.12 – 4
Trimethoprim-sulfamethoxazole	30.0	30.0	>4 / >4	≤0.5 – >4
Enterobacter cloacae **species complex (752)**				
Omadacycline			2 / 4	0.25 – >32
Tetracycline	85.6		2 / 16	0.5 – >16
Tigecycline	99.2	94.9	0.25 / 0.5	0.12 / 4
Ceftriaxone	67.2	67.2	0.25 / >8	≤0.06 – >8
Piperacillin-tazobactam	80.0	76.2	2 / 64	≤0.5 – >64
Levofloxacin	93.0	87.5	≤0.03 / 1	≤0.03 – >4
Gentamicin	92.7	92.0	0.25 / 1	0.12 – >8
Amikacin	99.2	98.8	1 / 2	0.5 – >32
Cefepime	88.0	82.2	≤0.12 / 4	≤0.12 – >16

Organism group (no. tested) antimicrobial agent	CLSI[a] %S	EUCAST[a] %S	$MIC_{50/90}$ (mg/L)	MIC range (mg/L)
Ceftazidime	72.1	69.1	0.5 / >32	0.06 – >32
Imipenem	97.3	98.3	0.25 / 0.5	≤0.12 – >8
Trimethoprim-sulfamethoxazole	84.4	84.4	≤0.5 / >4	≤0.5 – >4
**Ceftazidime-susceptible *Enterobacter cloacae* species complex (542)**				
Omadacycline			2 / 4	0.5 – >32
Tetracycline	92.4		2 / 4	0.5 – >16
Tigecycline	99.3	95.6	0.25 / 0.5	0.12 / 4
Ceftriaxone	93.1	93.1	0.25 / 1	≤0.06 – >8
Piperacillin-tazobactam	98.0	97.0	2 / 4	≤0.5 – >64
Levofloxacin	98.3	94.8	≤0.03 / 0.25	≤0.03 – >4
Gentamicin	99.3	99.1	0.25 / 0.5	0.12 – >8
Amikacin	100.0	100.0	1 / 2	0.5 – 8
Cefepime	99.4	98.7	≤0.12 / 0.25	≤0.12 – 16
Imipenem	99.6	99.8	0.25 / 0.5	≤0.12 – >8
Trimethoprim-sulfamethoxazole	92.8	92.8	≤0.5 / ≤0.5	≤0.5 – >4
**Ceftazidime-NS *Enterobacter cloacae* species complex (210)**				
Omadacycline			2 / 4	0.25 – 32
Tetracycline	68.1		2 / >16	1 – >16
Tigecycline	99.0	93.3	0.5 / 1	0.12 / 4
Ceftriaxone	0.5	0.5	>8 / >8	1 – >8
Piperacillin-tazobactam	33.5	22.0	64 / >64	1 – >64
Levofloxacin	79.0	68.6	0.12 / >4	≤0.03 – >4

Organism group (no. tested) antimicrobial agent	CLSI[a] %S	EUCAST[a] %S	MIC50/90 (mg/L)	MIC range (mg/L)
Gentamicin	75.7	73.8	0.25 / >8	0.12 – >8
Amikacin	97.1	95.7	1 / 4	0.5 – >32
Cefepime	58.6	39.5	2 / >16	≤0.12 – >16
Ceftazidime	0.0	0.0	>32 / >32	8 – >32
Imipenem	91.4	94.3	0.25 / 1	≤0.12 – >8
Trimethoprim-sulfamethoxazole	62.9	62.9	≤0.5 / >4	≤0.5 – >4
**Tetracycline-resistant *Enterobacter cloacae* species complex (87)**				
Omadacycline			4 / 16	1 – 32
Tigecycline	95.4	77.0	0.5 / 2	0.12 – 4
Ceftriaxone	29.9	29.9	>8 / >8	≤0.06 – >8
Piperacillin-tazobactam	57.0	40.7	16 / >64	1 – >64
Levofloxacin	67.8	44.8	1 / >4	≤0.03 – >4
Gentamicin	60.9	59.8	0.5 / >8	0.12 – >8
Amikacin	94.3	94.3	1 / 4	0.5 – >32
Cefepime	55.2	42.5	2 / >16	≤0.12 – >16
Ceftazidime	33.3	28.7	32 / >32	0.12 – >32
Imipenem	92.0	95.4	0.25 / 1	≤0.12 – >8
Trimethoprim-sulfamethoxazole	32.2	32.2	>4 / >4	≤0.5 – >4
**Other *Enterobacter* spp. (250)**				
Omadacycline			1 / 4	0.5 – 16
Tetracycline	91.2		1 / 4	0.5 – >16
Tigecycline	99.6	94.4	0.25 / 0.5	0.12 – 4

Organism group (no. tested) antimicrobial agent	CLSI[a] %S	EUCAST[a] %S	$MIC_{50/90}$ (mg/L)	MIC range (mg/L)
Ceftriaxone	70.3	70.3	0.12 / >8	≤0.06 – >8
Piperacillin-tazobactam	75.6	69.6	4 / 64	≤0.5 – >64
Levofloxacin	98.0	95.6	0.06 / 0.5	≤0.03 – >4
Gentamicin	99.2	99.2	0.25 / 0.5	≤0.06 – >8
Amikacin	99.6	99.2	1 / 2	≤0.25 – >32
Cefepime	97.6	94.4	≤0.12 / 0.5	≤0.12 – >16
Ceftazidime	72.8	68.4	0.25 / >32	0.06 – >32
Imipenem	96.8	98.4	0.5 / 1	≤0.12 – 8
Trimethoprim-sulfamethoxazole	97.6	97.6	≤0.5 / ≤0.5	≤0.5 – >4
**Other tetracycline-resistant *Enterobacter* spp. (17)**				
Omadacycline			16 / 16	2 – 16
Tigecycline	94.1	29.4	2 / 2	0.5 – 4
Ceftriaxone	52.9	52.9	1 / >8	0.12 – >8
Piperacillin-tazobactam	47.1	17.6	32 / >64	≤0.5 – >64
Levofloxacin	82.4	70.6	0.5 / >4	0.12 – >4
Gentamicin	94.1	94.1	0.25 / 1	0.12 – >8
Amikacin	100.0	100.0	1 / 4	0.5 – 8
Cefepime	88.2	70.6	0.5 / >16	≤0.12 – >16
Ceftazidime	52.9	23.5	2 / >32	0.25 – >32
Imipenem	94.1	100.0	0.5 / 1	≤0.12 – 2
Trimethoprim-sulfamethoxazole	88.2	88.2	≤0.5 / >4	≤0.5 – >4
***Citrobacter* spp. (354)**				

Organism group (no. tested) antimicrobial agent	CLSI[a] %S	EUCAST[a] %S	$MIC_{50/90}$ (mg/L)	MIC range (mg/L)
Omadacycline			1 / 4	0.25 – 16
Tetracycline	91.0		1 / 4	0.5 – >16
Tigecycline	100.0	98.9	0.25 / 0.5	0.12 – 2
Ceftriaxone	85.3	85.3	0.12 / >8	≤0.06 – >8
Piperacillin-tazobactam	87.6	81.1	2 / 64	≤0.5 – >64
Levofloxacin	95.5	91.0	≤0.03 / 0.5	≤0.03 – >4
Gentamicin	96.9	96.3	0.5 / 1	0.12 – >8
Amikacin	99.7	99.4	2 / 2	≤0.25 – 32
Cefepime	96.3	92.9	≤0.12 / 1	≤0.12 – >16
Ceftazidime	86.4	84.2	0.25 / >32	0.06 – >32
Imipenem	97.7	98.6	0.25 / 1	≤0.12 – 8
Trimethoprim-sulfamethoxazole	93.5	93.5	≤0.5 / ≤0.5	≤0.5 – >4
**Tetracycline-resistant *Citrobacter* spp. (23)**				
Omadacycline			4 / 8	0.5 – 8
Tigecycline	100.0	95.7	0.5 / 1	0.12 – 2
Ceftriaxone	60.9	60.9	1 / >8	≤0.06 – >8
Piperacillin-tazobactam	69.6	52.2	8 / >64	2 – >64
Levofloxacin	52.2	43.5	1 / >4	≤0.03 – >4
Gentamicin	78.3	78.3	0.5 / >8	0.12 – >8
Amikacin	95.7	95.7	2 / 2	0.5 – 32
Cefepime	82.6	78.3	≤0.12 / 4	≤0.12 – >16
Ceftazidime	60.9	52.2	1 / >32	0.25 – >32

Organism group (no. tested)<br>antimicrobial agent	CLSI[a]<br>%S	EUCAST[a]<br>%S	$MIC_{50/90}$ (mg/L)	MIC range (mg/L)
Imipenem	87.0	91.3	0.5 / 2	≤0.12 – 4
Trimethoprim-sulfamethoxazole	34.8	34.8	>4 / >4	≤0.5 – >4
***Citrobacter freundii* species complex (187)**				
Omadacycline			1 / 4	0.5 – 16
Tetracycline	85.0		1 / >16	0.5 – >16
Tigecycline	100.0	97.9	0.25 / 0.5	0.12 – 2
Ceftriaxone	74.9	74.9	0.25 / >8	≤0.06 – >8
Piperacillin-tazobactam	77.5	72.2	2 / >64	≤0.5 – >64
Levofloxacin	91.4	83.4	0.06 / 2	≤0.03 – >4
Gentamicin	94.1	93.0	0.5 / 1	0.25 – >8
Amikacin	99.5	98.9	2 / 4	0.5 – 32
Cefepime	93.6	87.2	≤0.12 / 2	≤0.12 – >16
Ceftazidime	74.9	71.1	0.5 / >32	0.12 – >32
Imipenem	95.7	97.3	0.5 / 1	≤0.12 – 8
Trimethoprim-sulfamethoxazole	88.8	88.8	≤0.5 / >4	≤0.5 – >4
**Tetracycline-resistant *Citrobacter freundii* species complex (20)**				
Omadacycline			4 / 8	0.5 – 8
Tigecycline	100.0	95.0	0.5 / 1	0.12 – 2
Ceftriaxone	60.0	60.0	1 / >8	≤0.06 – >8
Piperacillin-tazobactam	65.0	45.0	16 / >64	2 – >64
Levofloxacin	45.0	35.0	4 / >4	≤0.03 – >4
Gentamicin	75.0	75.0	0.5 / >8	0.25 – >8

Organism group (no. tested) antimicrobial agent	CLSI[a] %S	EUCAST[a] %S	$MIC_{50/90}$ (mg/L)	MIC range (mg/L)
Amikacin	95.0	95.0	2 / 2	0.5 – 32
Cefepime	80.0	75.0	≤0.12 / 4	≤0.12 – >16
Ceftazidime	55.0	50.0	1 / >32	0.25 – >32
Imipenem	85.0	90.0	0.5 / 2	≤0.12 – 4
Trimethoprim-sulfamethoxazole	35.0	35.0	>4 / >4	≤0.5 – >4
***Proteus mirabilis* (463)**				
Omadacycline			16 / >32	2 – >32
Tetracycline	1.1		>16 / >16	1 – >16
Tigecycline	69.8	26.3	2 / 4	0.25 – 8
Ceftriaxone	90.9	90.9	≤0.06 / 0.12	≤0.06 – >8
Piperacillin-tazobactam	99.6	99.4	≤0.5 / 1	≤0.5 – >64
Levofloxacin	80.1	67.4	0.06 / >4	≤0.03 – >4
Gentamicin	85.5	84.0	1 / >8	0.25 – >8
Amikacin	98.7	98.5	4 / 4	0.5 – >32
Aztreonam	98.5	96.5	≤0.03 / 0.06	≤0.03 – >16
Cefepime	93.7	92.2	≤0.12 / 0.25	≤0.12 – >16
Ceftazidime	96.8	94.0	0.06 / 0.25	0.03 – >32
Imipenem	33.3	81.9	2 / 4	≤0.12 – >8
Trimethoprim-sulfamethoxazole	69.7	69.7	≤0.5 / >4	≤0.5 – >4
**Tetracycline-resistant *Proteus mirabilis* (458)**				
Omadacycline			16 / >32	2 – >32
Tigecycline	69.4	26.0	2 / 4	0.25 - 8

Organism group (no. tested) antimicrobial agent	CLSI[a] %S	EUCAST[a] %S	$MIC_{50/90}$ (mg/L)	MIC range (mg/L)
Ceftriaxone	90.8	90.8	≤0.06 / 0.12	≤0.06 – >8
Piperacillin-tazobactam	99.6	99.3	≤0.5 / 1	≤0.5 – >64
Levofloxacin	80.1	67.5	0.06 / >4	≤0.03 – >4
Gentamicin	85.4	84.1	1 / >8	0.25 – >8
Amikacin	98.7	98.5	4 / 4	0.5 – >32
Aztreonam	98.5	96.5	≤0.03 / 0.06	≤0.03 – >16
Cefepime	93.7	92.1	≤0.12 / 0.25	≤0.12 – >16
Ceftazidime	96.7	93.9	0.06 / 0.25	0.03 – >32
Imipenem	33.2	81.9	2 / 4	≤0.12 – >8
Trimethoprim-sulfamethoxazole	69.6	69.6	≤0.5 / >4	≤0.5 – >4
**Indole-positive *Proteus* spp. (317)**				
Omadacycline			8 / 32	0.5 – >32
Tetracycline	38.2		16 / >16	0.5 – >16
Tigecycline	97.2	81.7	1 / 2	0.12 - 8
Ceftriaxone	79.8	79.8	≤0.06 / 8	≤0.06 – >8
Piperacillin-tazobactam	98.7	97.8	≤0.5 / 2	≤0.5 – >64
Levofloxacin	83.6	74.1	0.06 / >4	≤0.03 – >4
Gentamicin	88.3	85.2	0.5 / 8	0.12 – >8
Amikacin	99.1	99.1	2 / 4	≤0.25 – >32
Cefepime	97.2	95.9	≤0.12 / 0.25	≤0.12 – >16
Ceftazidime	89.6	79.5	0.25 / 8	≤0.015 – >32
Imipenem	42.0	97.2	2 / 2	≤0.12 – 8

Organism group (no. tested) antimicrobial agent	CLSI[a] %S	EUCAST[a] %S	$MIC_{50/90}$ (mg/L)	MIC range (mg/L)
Trimethoprim-sulfamethoxazole	79.8	79.8	≤0.5 / >4	≤0.5 – >4
**Tetracycline-resistant indole-positive *Proteus* spp. (169)**				
Omadacycline			8 / 32	0.5 – >32
Tigecycline	95.3	75.7	1 / 2	0.12 – 8
Ceftriaxone	77.5	77.5	≤0.06 / >8	≤0.06 – >8
Piperacillin-tazobactam	98.2	97.0	≤0.5 / 2	≤0.5 – >64
Levofloxacin	74.6	59.2	0.12 / >4	≤0.03 – >4
Gentamicin	81.1	75.1	1 / >8	0.12 – >8
Amikacin	98.2	98.2	2 / 4	≤0.25 – >32
Cefepime	95.3	92.9	≤0.12 / 0.5	≤0.12 – >16
Ceftazidime	87.0	73.4	0.25 / 8	0.03 – >32
Imipenem	52.7	97.6	1 / 2	≤0.12 – 8
Trimethoprim-sulfamethoxazole	68.0	68.0	≤0.5 / >4	≤0.5 – >4
***Serratia marcescens* (364)**				
Omadacycline			4 / 8	0.5 – >32
Tetracycline	11.0		>16 / >16	1 – >1
Tigecycline	99.2	92.3	1 / 1	0.12 – 8
Ceftriaxone	87.1	87.1	0.5 / 2	≤0.06 – >8
Piperacillin-tazobactam	95.9	92.9	2 / 8	≤0.5 – >64
Levofloxacin	95.9	87.6	0.12 / 1	≤0.03 – >4
Gentamicin	97.8	97.3	0.5 / 1	0.25 – >8
Amikacin	99.7	99.2	2 / 4	0.5 – 32

Organism group (no. tested) antimicrobial agent	CLSI[a] %S	EUCAST[a] %S	$MIC_{50/90}$ (mg/L)	MIC range (mg/L)
Cefepime	96.7	96.2	≤0.12 / 0.5	≤0.12 – >16
Ceftazidime	97.5	95.9	0.25 / 0.5	0.06 – >32
Imipenem	96.7	99.2	0.5 / 1	≤0.12 – >8
Trimethoprim-sulfamethoxazole	96.2	96.2	≤0.5 / 1	≤0.5 – >4
**Tetracycline-resistant *Serratia marcescens* (240)**				
Omadacycline			4 / 8	1 – >32
Tigecycline	98.8	89.6	1 / 2	0.25 – 8
Ceftriaxone	90.0	90.0	0.25 / 1	≤0.06 – >8
Piperacillin-tazobactam	97.1	95.0	2 / 8	≤0.5 – >64
Levofloxacin	94.2	85.4	0.12 / 1	≤0.03 – >4
Gentamicin	97.5	97.1	0.5 / 1	0.25 – >8
Amikacin	100.0	99.2	2 / 4	0.5 – 16
Cefepime	96.2	95.8	≤0.12 / 0.5	≤0.12 – >16
Ceftazidime	97.5	95.8	0.25 / 0.5	0.06 – >32
Imipenem	97.9	100.0	0.5 / 1	≤0.12 – 2
Trimethoprim-sulfamethoxazole	94.2	94.2	≤0.5 / 2	≤0.5 – >4
***Acinetobacter baumannii* (441)**				
Omadacycline			4 / 8	0.06 – >32
Tetracycline	26.8		>16 / >16	0.5 – >16
Tigecycline			2 / 4	≤0.06 - >8
Piperacillin-tazobactam	23.4		>64 / >64	≤0.5 – >64
Levofloxacin	25.4	24.0	>4 / >4	≤0.03 – >4

Organism group (no. tested) antimicrobial agent	CLSI[a] %S	EUCAST[a] %S	$MIC_{50/90}$ (mg/L)	MIC range (mg/L)
Gentamicin	31.1	31.1	>8 / >8	0.12 – >8
Amikacin	38.5	35.8	>32 / >32	0.5 – >32
Cefepime	24.3		>16 / >16	0.5 – >16
Imipenem	32.0	32.0	>8 / >8	≤0.12 – >8
Trimethoprim-sulfamethoxazole	44.0	44.0	>4 / >4	≤0.5 – >4
**Tetracycline-resistant *Acinetobacter baumannii* (293)**				
Omadacycline			4 / 8	0.25 – >32
Tigecycline			2 / 4	0.25 - >8
Piperacillin-tazobactam	2.4		>64 / >64	≤0.5 – >64
Levofloxacin	2.4	1.7	>4 / >4	0.12 – >4
Gentamicin	11.9	11.9	>8 / >8	0.25 – >8
Amikacin	19.1	15.7	>32 / >32	0.5 – >32
Cefepime	3.1		>16 / >16	2 – >16
Imipenem	8.2	8.2	>8 / >8	≤0.12 – >8
Trimethoprim-sulfamethoxazole	25.9	25.9	>4 / >4	≤0.5 – >4
***Stenotrophomonas maltophilia* (315)**				
Omadacycline			2 / 8	0.25 – >32
Tetracycline			16 / >16	2 – >16
Tigecycline			1 / 2	0.12 – 8
Piperacillin-tazobactam			>64 / >64	16 – >64
Levofloxacin	83.2		1 / >4	0.25 – >4
Cefepime			>16 / >16	2 – >16

Organism group (no. tested) antimicrobial agent	CLSI[a] %S	EUCAST[a] %S	$MIC_{50/90}$ (mg/L)	MIC range (mg/L)
Ceftazidime	20.0		>32 / >32	1 – >32
Trimethoprim-sulfamethoxazole	95.6	96.8	≤0.5 / 1	≤0.5 – >4
***Haemophilus influenzae* (803)**				
Omadacycline			1 / 1	0.12 – 16
Tetracycline	99.8	99.5	0.5 / 1	≤0.06 – >8
Tigecycline	96.1		0.12 / 0.25	0.06 - 1
Ceftriaxone	100.0	99.5	0.004 / 0.015	≤0.001 – 0.5
Amoxicillin-clavulanic acid	99.4	93.9	0.5 / 2	0.12 – >8
Levofloxacin	99.8	98.3	0.015 / 0.03	0.008 – >2
Ampicillin	65.5	65.5[b]	0.5 / >8	0.12 – >8
Azithromycin	99.0	0.9	0.5 / 1	0.12 – >32
Moxifloxacin	99.8	98.9	0.03 / 0.06	0.008 – >1
Trimethoprim-sulfamethoxazole	65.1	65.1	0.12 / >4	≤0.06 – >4
**β-lactamase-positive *Haemophilus influenzae* (201)**				
Omadacycline			1 / 1	0.25 – 4
Tetracycline	100.0	100.0	0.5 / 0.5	0.25 – 1
Tigecycline	93.5		0.12 / 0.25	0.06 – 1
Ceftriaxone	100.0	99.0	0.004 / 0.015	0.002 – 0.5
Amoxicillin-clavulanic acid	99.0	92.0	1 / 2	0.5 – >8
Levofloxacin	100.0	97.5	0.015 / 0.03	0.008 – 0.25
Ampicillin	0.0	0.0[b]	>8 / >8	4 – >8
Azithromycin	98.0	0.5	0.5 / 1	0.12 – >32

Organism group (no. tested)<br>antimicrobial agent	CLSI[a] %S	EUCAST[a] %S	$MIC_{50/90}$ (mg/L)	MIC range (mg/L)
Moxifloxacin	100.0	99.0	0.03 / 0.06	0.008 – 0.5
Trimethoprim-sulfamethoxazole	62.7	62.7	0.12 / >4	≤0.06 – >4
**β-lactamase-negative *Haemophilus influenzae* (602)**				
Omadacycline			1 / 1	0.12 – 16
Tetracycline	99.7	99.3	0.5 / 1	≤0.06 – >8
Tigecycline	97.0		0.12 / 0.25	0.06 – 1
Ceftriaxone	100.0	99.7	0.004 / 0.015	≤0.001 – 0.5
Amoxicillin-clavulanic acid	99.5	94.5	0.5 / 2	0.12 – >8
Levofloxacin	99.7	98.5	0.015 / 0.03	0.008 – >2
Ampicillin	87.4	87.4[b]	0.25 / 2	0.12 – 8
Azithromycin	99.3	1.0	1 / 1	0.12 – >32
Moxifloxacin	99.7	98.8	0.03 / 0.06	0.008 – >1
Trimethoprim-sulfamethoxazole	65.9	65.9	0.12 / >4	≤0.06 – >4
***Haemophilus parainfluenzae* (18)**				
Omadacycline			1 / 2	0.5 – 16
Tetracycline	77.8	77.8	0.5 / >8	0.25 – >8
Tigecycline			0.25 / 0.5	0.12 – 1
Ceftriaxone	100.0	94.4	0.008 / 0.12	≤0.001 – 0.25
Amoxicillin-clavulanic acid	100.0	94.4	0.5 / 2	0.12 – 4
Levofloxacin	88.9	83.3	0.03 / >2	0.015 – >2
Ampicillin	77.8	77.8[b]	0.5 / 4	0.12 – 8
Azithromycin	100.0	5.6	1 / 4	0.12 – 4

Organism group (no. tested)<br>antimicrobial agent	CLSI[a] %S	EUCAST[a] %S	$MIC_{50/90}$ (mg/L)	MIC range (mg/L)
Moxifloxacin	88.9	66.7	0.06 / >1	0.015 – >1
Trimethoprim-sulfamethoxazole	77.8	77.8	≤0.06 / >4	≤0.06 – >4
***Moraxella catarrhalis* (408)**				
Omadacycline			0.25 / 0.25	0.06 – 0.5
Tetracycline	100.0	100.0	0.25 / 0.5	0.12 – 0.5
Tigecycline			0.06 / 0.06	≤0.015 – 0.12
Ceftriaxone	100.0	99.8	0.25 / 0.5	0.002 – 2
Amoxicillin-clavulanic acid	100.0	100.0	0.12 / 0.25	≤0.06 – 0.5
Levofloxacin	100.0	100.0	0.06 / 0.06	0.015 – 1
Azithromycin	99.7	99.7	0.015 / 0.03	0.008 – 1
Moxifloxacin		100.0	0.06 / 0.06	0.015 – 0.5
Penicillin			>4 / >4	≤0.03 – >4
Trimethoprim-sulfamethoxazole	96.6	96.6	0.12 / 0.25	≤0.06 – 2

[a] Criteria as published by CLSI [2017] and EUCAST [2017].

[b] β-lactamase test positive reported as resistant for penicillins without inhibitors..
